# Immune checkpoint inhibitor-induced diabetes mellitus in metastatic NSCLC: a case report with extended follow-up and management considerations

**DOI:** 10.3389/fimmu.2026.1874841

**Published:** 2026-07-17

**Authors:** Daniele Nova, Gabriele Giuseppe Pagliari, Sara Mambrito, Diego Luigi Cortinovis, Stefania Canova

**Affiliations:** 1 Medical Oncology Unit, Fondazione Istituto di Ricovero e Cura a Carattere Scientifico (IRCCS) San Gerardo dei Tintori, Monza, Italy; 2 Medicine and Surgery Department, Milano Bicocca University, Milan, Italy

**Keywords:** diabetes mellitus, immunocheck point inhibitors, immunorelated adverse events, non small cell lung cancer, pembrolizumab

## Abstract

Immune checkpoint inhibitors-induced diabetes mellitus (ICI-DM) is a rare but potentially life-threatening endocrine immune-related adverse event, often characterized by abrupt onset of insulin deficiency and frequent presentation with diabetic ketoacidosis and difficulty with daily management with the available therapies. Lung cancer patients represent a substantial proportion of reported cases, reflecting the widespread use of PD-1/PD-L1 inhibitors in thoracic oncology. We report on the case of an elderly patient with metastatic lung adenocarcinoma treated with pembrolizumab who developed severe DM requiring permanent insulin therapy and leading to treatment discontinuation. The patient was subsequently followed over a prolonged period, during which oncological disease remained under sustained control despite immunotherapy interruption. We describe the clinical course, diagnostic workup, and multidisciplinary management, and review current guideline recommendations addressing acute metabolic management, diabetic treatment, and decision-making regarding continuation of immunotherapy. This case highlights the complexity of managing ICI-DM in real-world clinical practice. The current guidelines may help in broad terms. Although guidelines have been published, they remain cursory. Nevertheless, therapeutic decisions should ultimately be individualized through close multidisciplinary collaboration.

## Introduction

Immune checkpoint inhibitors (ICI) targeting programmed cell death protein 1 (PD-1) and programmed death-ligand 1 (PD-L1) have significantly improved survival outcomes in patients with advanced or metastatic non-small cell lung cancer (NSCLC), particularly compared with platinum-based chemotherapy alone. Several pivotal clinical trials demonstrated substantial improvements in progression-free survival (PFS) and overall survival (OS). In the KEYNOTE-024 trial, pembrolizumab monotherapy in treatment-naïve NSCLC patients with a PD-L1 tumor proportion score (TPS) ≥50% significantly improved median OS compared with platinum-based chemotherapy (26.3 vs 13.4 months; HR 0.62) (1). Similarly, the KEYNOTE-189 and KEYNOTE-407 trials demonstrated significant survival benefits with the addition of pembrolizumab to platinum-based chemotherapy in non-squamous and squamous NSCLC, respectively, regardless of PD-L1 expression levels (2, 3). Comparable efficacy results have also been reported with other PD-1/PD-L1 inhibitors, including atezolizumab and nivolumab, across different treatment settings (4, 5). Nevertheless, the magnitude of clinical benefit may vary according to PD-L1 expression, histological subtype, smoking status, age, comorbidities, performance status, and other patient-related factors. Importantly, although older age is frequently associated with concerns regarding treatment tolerance, accumulating evidence suggests that immunotherapy is generally safe and effective in elderly patients with advanced NSCLC. A recent multicenter study by Tsukita et al. demonstrated that immunotherapy-based approaches, including chemoimmunotherapy, achieved favorable clinical outcomes in older adults without raising significant age-related safety concerns (6). Likewise, data from the Italian expanded access program showed that nivolumab maintained a manageable safety profile and meaningful clinical activity in elderly patients with advanced squamous NSCLC, supporting its use in this population (7). These findings indicate that chronological age alone should not represent a barrier to treatment with ICI, and that therapeutic decisions should instead be guided by a comprehensive assessment of patients’ functional status and comorbidities However, immune system activation may result in immune-related adverse events (irAEs), which can affect virtually any organ system, including the endocrine glands.

Among endocrine irAEs, immune checkpoint inhibitor-induced diabetes mellitus (ICI-DM) is rare, with a reported incidence ranging from 0.2% to 1.4% (8–10), but it represents one of the most severe endocrine toxicities due to its abrupt onset, frequent presentation with diabetic ketoacidosis (DKA), and irreversible insulin dependence (11, 12). Unlike other endocrine irAEs, recovery of endocrine function is exceptionally rare (9, 11, 12), and long-term metabolic complications may significantly impact patient quality of life and overall prognosis.

ICI-DM is characterized by rapid and often irreversible destruction of insulin-producing pancreatic β-cells, leading to absolute insulin deficiency. Although uncommon, lung cancer patients account for approximately 20-30% of reported cases (8), reflecting the widespread use of PD-1/PD-L1 inhibitors in thoracic oncology. Early recognition and appropriate management are essential to prevent life-threatening complications and to guide complex decisions regarding either continuation or discontinuation of immunotherapy.

We report a case of pembrolizumab-associated immune-mediated diabetes in an elderly patient with metastatic lung adenocarcinoma, with particular focus on diagnostic challenges, guideline-based and individualized management strategies, and long-term oncological outcomes following immunotherapy discontinuation.

## Case presentation

A 72-year-old male with a history of hypertension, benign prostatic hyperplasia, remote pulmonary tuberculosis, former heavy smoking and without known family history of diabetes, presented in April 2018 with persistent cough refractory to antibiotic therapy.

A thoracic CT scan revealed a 20 mm right upper lobe pulmonary nodule and an enlarged right hilar lymph node. Fluorodeoxyglucose positron emission tomography (18F-FDG-PET) demonstrated increased metabolic activity in both lesions, with no evidence of distant disease. Brain CT was negative. Transthoracic biopsy showed lung adenocarcinoma. Thus, the patient underwent right upper lobectomy with mediastinal lymphadenectomy in June 2018.

Histopathological examination confirmed lung adenocarcinoma with lymphovascular and perineural invasion, as well as nodal involvement. According to the 8^th^ edition the Tumor-Node-Metastasis (TNM) staging system, the pathological stage was pT2a pN2, corresponding to stage IIIA disease. Specifically, pT2a indicates a primary tumor measuring >3 cm but ≤4 cm in greatest dimension, while pN2 indicates ipsilateral mediastinal lymph node involvement. Lymph node metastases were identified in stations 4R (right lower paratracheal), 6 (para-aortic), and 11R (right interlobar), according to the International Association for the Study of Lung Cancer lymph node map. Molecular analysis was negative for EGFR, ALK, ROS1, and BRAF alterations, while PD-L1 had a tumor proportion score (TPS) of 80%.

Following multidisciplinary discussion, the patient received adjuvant chemotherapy with cisplatin and vinorelbine for 4 cycles, followed by mediastinal radiotherapy (50 Gy in 28 fractions) because of multi-station lymph node metastases. Follow-up imaging remained negative until May 2020, when CT scan showed brain and liver metastases. Brain MRI confirmed a solitary cerebral lesion, which was treated with stereotactic radiotherapy (SBRT), 21 Gy in 1 fraction. Systemic therapy with pembrolizumab (400 mg every 6 weeks) was initiated in August 2020.

The patient achieved a sustained partial response of both brain and hepatic lesions according to RECIST 1.1 criteria in about 10 weeks (**Figure 1**).

**Figure 1:**
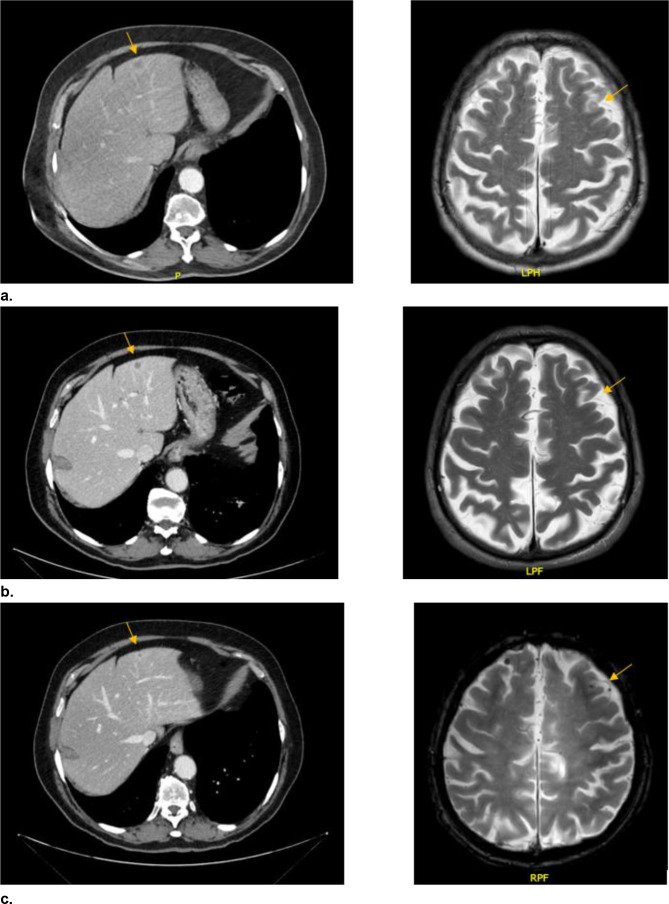
**(a)** Baseline CT scan and MRI scan showed a 13 mm single hepatic lesion in segment II and a solitary brain metastasis in left superior frontal area. **(b)** After 12 weeks CT scan and MRI showed a 9 mm single hepatic lesion in segment II and a focus enhancement in left superior frontal area. **(c)** After 24 weeks CT scan and MRI showed a 6 mm single hepatic lesion in segment II and the lesion in left superior frontal area is no longer detectable.

Pembrolizumab was initially well tolerated. In October 2021, after 10 treatment cycles, the patient reported metabolic symptoms including polyuria, polydipsia, and weight loss, without history of diabetes (glycemia value was 93 mg/dL before starting pembrolizumab). Laboratory evaluation revealed hyperglycemia (459 mg/dL). The patient was referred to the Emergency Department, where laboratory tests confirmed hyperglycemia (690 mg/dL), and showed preserved renal function, normal values of electrolytes with metabolic acidosis – pH 7, 31, lactate 3 mmol/L, bicarbonate 18 mEq/L. Therefore, the patient was hospitalized and started a continuous infusion of insulin in association with saline solution, in order to adjust glucose level, blood pH and balance electrolytes, in particular potassium serum level, which remained in range during insulin infusion. Simultaneously, he had further laboratory investigations to define the nature of hyperglycemia. Laboratory findings suggested impaired endogenous insulin secretion, with serum insulin level of 2.5 microUI/mL (reference value > 2.6 microUI/mL) and low-borderline C-peptide value of 1.1 ng/mL (reference value >1.1 ng/mL), consistent with evolving insulin deficiency. Moreover, autoantibodies against glutamic acid decarboxylase (GADA) and islet cell antibodies (ICA) were negative. Finally, other causes of hyperglycemia were excluded, like infectious diseases, medications (e.g. corticosteroid, atypical antipsychotics), other endocrinopathies (e.g. Cushing), and pancreatic pathologies. Therefore, the clinical diagnosis was attributed to ICI-DM. HbA1c values at presentation were unfortunately unavailable, limiting further characterization of the chronicity of hyperglycemia. Gradually, a basal-bolus insulin regimen was initiated with a progressive glycemic control, according to an insulin optimization. After 5 days of hospitalization the patient was discharged in good clinical conditions. A multidisciplinary discussion with medical oncologists and endocrinologists was conducted to define a potential pembrolizumab restart. However, considering advanced age, comorbidities, high risk of glycemic decompensation and time since last administration of pembrolizumab, clinicians agreed on its discontinuation.

During follow-up, the patient remained permanently insulin-dependent, with no recovery of endogenous insulin secretion. After discontinuation of pembrolizumab, diabetes remained under control, with no further episodes of glycemic decompensation. Long-term oncological surveillance demonstrated a disease control until December 2025, when thoracic–abdominal CT showed no evidence of systemic progression, while brain MRI revealed intracranial disease progression, with the appearance of a new lesion in the left postcentral gyrus, for which he subsequently underwent SBRT, 21 Gy in 1 fraction. Blood glucose values ​​persisted within the normal range despite the initiation of brain metastases prophylactic therapy with dexamethasone. The patient remains clinically stable under oncological and endocrinological follow-up (**Figure 2**).

**Figure 2:**
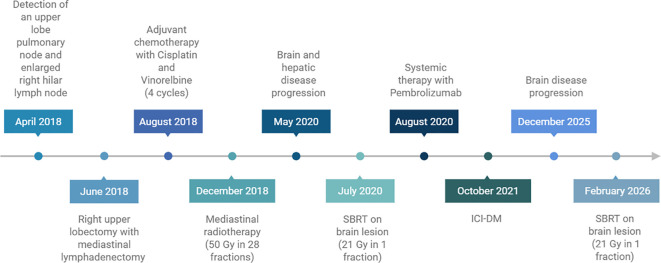
Timeline of relevant clinical events, (SBRT, Stereotactic Body Radiotherapy; ICI-DM, Immune Checkpoint Inhibitors-induced Diabetes Mellitus).

## Discussion

ICI-DM is an uncommon but increasingly recognized irAE associated with PD-1/PD-L1 blockade. Although its reported incidence ranges between 0.2% and 1.4% (8–10), its clinical relevance is disproportionate to its frequency due to the abrupt onset, frequent presentation with DKA, and the requirement for lifelong insulin therapy. Lung cancer patients represent a substantial proportion of reported cases, reflecting the widespread and prolonged use of ICI in thoracic oncology.

The present case displays several hallmark features of ICI-DM described in the literature, including delayed onset after several months of immunotherapy, rapid progression to insulin deficiency, absence of islet autoantibodies, and permanent insulin dependence. While glutamic acid decarboxylase antibodies (GADA) are detected in approximately 40-50% of ICI-DM cases (11) their absence, as observed in our case, does not exclude the diagnosis. The low-borderline C-peptide levels, together with persistent insulin dependence, support a rapidly evolving insulin-deficient state associated with immune checkpoint inhibitor therapy. However, given the detectable C-peptide level and absence of islet autoantibodies, neither a definitive diagnosis of fulminant type 1 diabetes nor a partial insulin resistance can be established. Current evidence suggests that immune checkpoint blockade triggers a T cell-mediated immune attack against pancreatic β-cells, leading to irreversible endocrine dysfunction (10, 12, 13).

An important clinical aspect highlighted by this case is the time of onset of ICI-DM. Although most cases occur within the first months of treatment, delayed presentations have also been increasingly described, including after ICIs discontinuation (8, 14, 15). This underscores the need for long-term metabolic vigilance, even in patients with good tolerance to immunotherapy for an extended period.

Unlike several other immune-related adverse events, management of ICI-DM primarily relies on insulin replacement therapy and metabolic stabilization. All guidelines emphasize the importance of baseline glucose and HbA1c assessment prior to initiation of ICI, regular glucose monitoring during treatment, and immediate evaluation in case of symptoms of hyperglycemia (16–18).

Serum glucose monitoring should be performed at each cycle during the first 12 weeks, considering more frequent checks when glucose values are borderline (16, 19). Patient education is mandatory, as they have to be informed about possible signs or symptoms, like polyuria/polydipsia, nausea, fatigue, confusion. In case of serum glucose >160 mg/dL or suspicious symptoms, diagnostic labs (glucose, ketones, electrolytes, creatinine, C-peptide, blood gas analysis) and endocrinologist consultation should be rapidly obtained. Acute management focuses on prompt recognition of hyperglycemia, DKA and rapid initiation of insulin therapy, often requiring hospitalization and multidisciplinary management, including endocrinologists and intensive care specialists in severe cases. Once established, insulin dependence is typically permanent, and recovery of endogenous insulin secretion is not expected.

One of the major challenges in the management of ICI-DM is the decision regarding continuation, withholding, or permanent discontinuation of immunotherapy. Current guidelines provide a general framework but allow for clinical discretion. American Society of Clinical Oncology (ASCO), European Society For Medical Oncology (ESMO), and Society for Immunotherapy of Cancer (SITC) guidelines suggest that ICI may be resumed in selected patients once glycemic control is achieved, particularly in those deriving substantial oncological benefit and without recurrent metabolic instability. However, permanent discontinuation may be appropriate in frail patients, those with severe or recurrent metabolic decompensation, or when safe glycemic control cannot be reliably maintained (16–18). Notably, while ESMO, ASCO, and SITC guidelines provide largely consistent recommendations regarding acute metabolic management, slight differences exist in their approach to immunotherapy rechallenge, further supporting the need for individualized clinical decision-making **Figure 3**.

**Figure 3:**
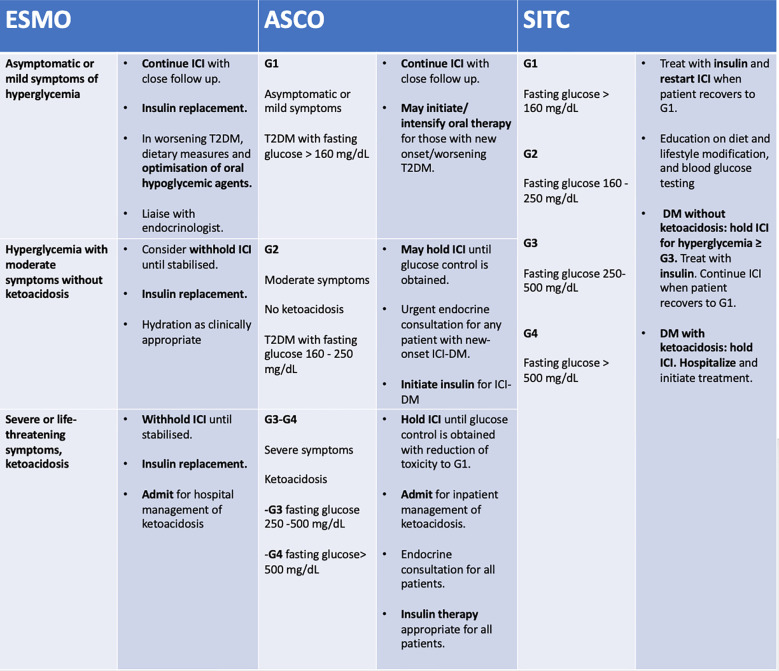
Comparative table of the ESMO, ASCO, and SITC guidelines on the classification and management of ICI-DM (16–18). ESMO, European Society For Medical Oncology; ASCO, American Society of Clinical Oncology; SITC, Society for Immunotherapy of Cancer; ICI, Immune Checkpoint Inhibitors; T2DM, Type 2 Diabetes Mellitus; DM, Diabetes Mellitus.

Despite ongoing oncological response and the therapeutic setting, which is a first line treatment, pembrolizumab was suspended indefinitely in our case as per a decision defined by multidisciplinary discussion with endocrinologists (20). The patient was in fact deemed to be exposed to an elevated risk of glycemic decompensation due to advanced age, comorbidities and above all the severity of diabetes insurgence. Moreover, it is indeed crucial that the patient be properly trained in handling both the insulin therapy and the glycemic control. Educational path should be complemented with dietary and physical advice too (21).

This clinical report adds to emerging evidence suggesting that durable responses may persist after ICI interruption due to irAEs. This observation is consistent with previous reports suggesting that durable oncological responses may persist in selected patients after ICI interruption, although the relationship between immune-related adverse events and long-term oncological benefit remains incompletely understood. Moreover, no causal relationship can be inferred from a single case report (22–24).

While previously published case reports have largely focused on the acute presentation and metabolic management of ICI-induced diabetes, the present case provides insight into long-term oncological outcomes and real-world decision-making regarding immunotherapy discontinuation. It underscores that, although existing guidelines offer an essential framework for diagnosis and treatment, management of ICI-DM ultimately requires individualized, multidisciplinary decision-making tailored to patient-specific factors, particularly in either elderly or frail individuals.

## Conclusions

ICI-DM represents a rare but severe complication of immunotherapy in lung cancer patients. Early recognition, appropriate diagnostic evaluation, and prompt initiation of insulin therapy are essential to prevent life-threatening complications. Permanent insulin dependence is commonly observed and requires long-term endocrinological management.

This case illustrates the complexity of managing ICI-DM in real-world clinical practice, particularly regarding decisions on immunotherapy discontinuation. While current guidelines provide an essential framework, management must be individualized within a multidisciplinary team, especially in either elderly or frail patients. Long-term follow-up data from this case suggest that durable oncological control may persist despite permanent immunotherapy discontinuation, reinforcing the importance of patient-centered decision-making.

## Data Availability

The datasets presented in this article are not readily available because there are not datasets. It is a clinical case report. Clinical data are available by request. Requests to access the datasets should be directed to daniele.nova@irccs-sangerardo.it.
